# K_x_Co_1.5−0.5x_Fe(CN)_6_/rGO with Dual−Active Sodium Ion Storage Site as Superior Anode for Sodium Ion Battery

**DOI:** 10.3390/nano13020264

**Published:** 2023-01-07

**Authors:** Gang Zhou, Mincong Fan, Lei Wang, Xianglin Li, Danqing Liu, Feng Gao

**Affiliations:** 1 School of Environment and Civil Engineering, DongGuan University of Technology, Dongguan 523808, China; 2 College of Materials Science and Engineering, Shenzhen University, Shenzhen 518061, China; 3 School of Physics and Chemistry, Hunan First Normal University, Changsha 410205, China

**Keywords:** prussian blue analogues, dual−active, anode, sodium ion battery

## Abstract

The unique and open large frame structures of prussian blue analogues (PBA) enables it for accommodating a large number of cations (Na^+^, K^+^, Ca^2+^, etc.), thus, PBA are considered as promising electrode materials for the rechargeable battery. However, due to the chemical composition, there are still many alkaline metal ions in the gap within the framework, which puts multivalent metals in PBA in a low valence state and affects the sodium storage performance. To improve the valence of metal ions in PBA materials, precursors prepared by co−precipitation method and hydrothermal method are used to synthesis K_x_Co_1.5−0.5x_Fe(CN)_6_ through further chemical oxidation. Through the introducing of reduced graphene oxide (rGO) with excellent conductivity by a simple physical mixing method, the cycle stability and rate performance of the PBA material can be further improved. The K_0.5_Co_1.2_Fe(CN)_6_·2H_2_O/rGO anode prepared with 2 h hydrothermal time and further chemical oxidation, named as KCoHCP−H2−EK/rGO, exhibits a super electrochemical performance, delivering initial charge/discharge capacities of 846.7/1445.0 mAh·g^−1^, and a capacity retention of 58.2% after 100 cycles at a current density of 100 mA·g^−1^. The KCoHCP−H2−EK/rGO outstanding electrochemical behaviors are attributed to the unique dual−active site structure properties and the improved surface conductance of materials by rGO components.

## 1. Introduction

Owing to the low−cost and natural abundance of sodium, sodium−ion batteries (SIBs) are considered as an alternative battery for lithium−ion batteries (LIBs), which has attracted extensive research attention recently [1,2,3]. Although the energy density of SIBs is not as high as that of LIBs, given the current high price of lithium carbonate, SIBs have broad application prospects in areas that do not require high energy density, such as smart grid energy storage [4,5,6,7], base station energy storage [8,9,10], and low−speed vehicles power [11,12,13,14], etc. However, the optimization and design of suitable sodium storage electrode materials are still the key challenges.

Due to its unique open macroframe structure, Prussian blue/Prussian blue analogs (PB/PBA) are capable for accommodating a large number of cations (Na^+^, K^+^, Ca^2+^, Mg^2+^, Sr^2+^, and Ba^2+^, etc.), without structural collapse when ions are deintercalated, which make them considered as promising electrode materials for secondary batteries [15,16,17,18,19]. Up to date, as an effective sodium source material, PB/PBA has been widely used as a cathode material for SIBs [20,21,22]. For example, Wu et al. proposed a facile citrate−assisted synthesis of low−defect and highly crystalline Na_2_CoFe(CN)_6_ nanocrystals, and the as synthesized Na_2_CoFe(CN)_6_ exhibited a highly reversible two Na^+^ reaction with a high capacity of 150 mAh·g^−1^ and an excellent long−term cycling performance of about 90% capacity retention over 200 cycles [23]. Based on current research, the capacity of the PB/PBA as SIBs cathode is very close to its theoretical capacity value. In order to further improve the sodium storage capacity of PB/PBAs, researchers have developed the application of PB/PBA as anode materials [24]. Chen et al. synthesized K_0.01_Cr_3_[Cr(CN)_6_]_2_ (CrCr−PBA) as aqueous SIBs anode material by coprecipitation method, and the energy storage capacity of CrCr−PBA was found to be 108.2 mAh·g^−1^. It has been reported that the specific discharge capacity of the fabricated CrCr−PBA material decreased slightly to 93.4% of the initial value with increasing cycle time [25]. Piernas−Munoz et al. reported that K_0.88_Fe^III^_1.04_[Fe^II^(CN)_6_]·yH_2_O (PB) can be simply synthesized through a solvothermal method and it can be used as anode of LIBs. The PB anodes has been showed reversible capacity up to 400 mAh·g^−1^ at 8.75 mA·g^−1^ [26]. Nie et al. reported the synthesis of Co_3_[Co(CN)_6_]_2_·nH_2_O nanocubes by precipitation method and the as synthesized nanocubes can be used as the main framework of LIBS cathode. It has been found that the Co_3_[Co(CN)_6_]_2_ nanocubes negative electrode can provide 299.1 mAh·g^−1^ reversible capacity and high coulomb efficiency [27]. At present, the research of PB/PBS cathode materials is still in the exploratory stage, and there is still a gap in capacity compared with other cathode materials. However, there are still many alkaline metal ions in the framework because of the chemical composition effect, which will affect its capacity. If the alkaline metal ions in the gap can be removed without structural collapse, the multivalent metals in PB/PBA will effectively improve the performance of the multi−electron energy storage.

In this work, the precursors of KCoFe(CN)_6_ are prepared by coprecipitation and hydrothermal methods, respectively. Then, the KCoFe(CN)_6_ precursors are transformed into potassium−eliminated CoFe(CN)_6_ by chemical oxidation method (inspired by the work on the preparation of FePO_4_ [28,29] and the research work on Li^+^ extraction from LiMn_2_O_4_ [30]. The as synthesized CoFe(CN)_6_ are used as anode materials of SIBs. Both of Co and Fe ions exhibit two valence states of +2 and +3 in the discharge/charge state, which provides the double−active sites for PBA anodes. The two−electron energy storage process leads to a better sodium storage performance. The reduced graphene oxide (rGO) with excellent conductivity [31,32] is introduced into the negative electrode material system forms a PBA/rGO composites anodes for sodium battery application. It has been found an improved cycle stability and magnification performance of SIBs with PBA anode.

## 2. Experimental Section

### 2.1. Synthesis of KCoFe(CN)_6_ (KCoHCP)

All chemicals are of analytical grade and used without further purification. The KCoFe(CN)_6_ sample was synthesized using a co−precipitation route at room temperature [33,34]. Firstly, CoCl_2_·H_2_O (475.9 mg) and trisodium citrate dihydrate (516.1 mg) were dissolved in H_2_O (80 mL) under vigorous stirring to give a transparent and homogeneous solution (solution A). Secondly, K_3_Fe(CN)_6_ (658.5 mg) was dissolved in H_2_O (10 mL) under agitated stirring to obtain a transparent solution (solution B). Thirdly, after stirring solution A for 1 h, solution B was added to solution A slowly and regularly, then the mixed solution was agitated stirring at room temperature for 12 h. Finally, the resulting precipitate was collected by centrifugation, washed several times with deionized water and anhydrous ethanol, and then dried at 60 °C overnight. For comparison, the mixture of solution A and solution B was immediately transferred into a Teflon−lined stainless steel autoclave and maintained in the oven at 200 °C for 1, 2, 4, and 12 h respectively while other experiment parameters were kept unchanged. The obtained samples were named KCoHCP−CP and KCoHCP−Hx (x are hydrothermal reaction time, x = 1, 2, 4, and 12).

### 2.2. Synthesis of K_x_Co_1.5−0.5x_Fe(CN)_6_ (KCoHCP−EK)

The K_x_Co_1.5−0.5x_Fe(CN)_6_ is prepared by the chemical method of extracting K atoms. For the preparation of K_x_Co_1.5−0.5x_HCP, the obtained solid KCoHCP−CP and KCoHCP−H particles (500 mg) and Na_2_S_2_O_8_ (2.38 g) were respectively added to H_2_O (50 mL) in beaker under magnetic stirring at 80 °C for 12 h. The obtained samples were named KCoHCP−CP−EK and KCoHCP−Hx−EK.

### 2.3. Synthesis of KCoHCP−EK/rGO

Graphene oxide (GO) was synthesized using modified Hummers method [35,36]. 0.5 g of KCoHCP were dissolved in 50 mL deionized water (solution A) and Go solutions of 2 mg/mL and 1 mg/mL respectively. Solution A was sonicated for 30 min and stirred for 30 min separately. Then CTAB (0.2 g) was stirred intensely into A and continue stirring for 2 h, which was named as solution B. After stirring solution B for 2 h, Go solution was added to solution B slowly and regularly, then the mixed solution was agitated stirring at room temperature for 2 h. Then, mixed solution was watered with deionized water and ethanol multiple times. The washed sample was redispersed in 50 mL of water and add 400 μL of 50% hydrazine hydrate solution and stirred in a water bath at 95 °C for 1.5 h. Finally, the resulting precipitate was collected by centrifugation, washed several times with distill water and anhydrous ethanol, and then dried at 60 °C for overnight.

### 2.4. Measurements

The active materials were mixed with super P and LA133 binder at a weight ratio of 90:5:5 in H_2_O and then the obtained slurry covered on the Cu foils. The electrode film was subsequently pressed and dried in a vacuum drying oven for 12 h at 120 °C. The electrochemical properties of all anode materials were measured using coin−type cells (CR2032) assembled in a dry glove box filled with pure argon with sodium metal as the anode and Celgard 2500 polypropylene membrane as separator. The electrolyte was 1.0 M NaPF_6_ in a solution composed of EC, DMC, and EMC (with volume ratio of 1:1:1) with 5% FEC. The coin cells of galvanostatic charge/discharge were investigated at various rates with the potential range of 0.05–3.0 V at 25 °C, and coin cells were cycled at 0.1 A g^−1^ rate between 0.05 and 3.0 V using a Land Test System (CT2001A). Cyclic voltammetry (CV) measurements were performed between 0.05 V and 3.0 V with a sweeping speed of 0.1 mV s^−1^ on an electrochemical workstation (IVIUM). Electrochemical impedance spectroscopy (EIS) was characterized by the same electrochemical workstation for all the samples in the frequency range of 0.01 Hz–0.1 MHz with a voltage amplitude of 5 mV.

Powder X-ray diffraction (XRD; Escalab 250xi; Thermo Scientific; Waltham, MA, USA) was used to characterize the crystalline phase of samples with Cu Kα radiation in the 2 θ range of 10–80°.

Elemental analysis for metals (K, Co, and Fe) was conducted by Inductively coupled plasma−optical emission spectrometry (ICP−OES 730; Agilent; Santa Clara, CA, USA). The thermal stability of the as−prepared sample was determined by thermogravimetric analysis (TGA, STA 449 F3 Jupiter; NETZSCH; Selb, Germany) under N_2_ flow with a heating rate of 10 °C min^−1^. And all samples were observed via field emission scanning electron microscope (SEM; Regulus8100; Hitachi; Hitachi, Japan). The surface chemical valence states of the samples were analyzed by X-ray photoelectron spectroscopy (XPS, TEscalab 250xi; Thermo Scientific; Waltham, MA, USA). The Fourier transform infrared spectra and Raman spectra were carried on Fourier transforming infrared spectrometer (FT−IR, Thermo Nicolet iS5; Thermo Scientific; Waltham, MA, USA) and laser Raman spectrometer (Raman, Thermo Fisher DXRxi; Thermo Scientific; Waltham, MA, USA), respectively.

## 3. Results and Discussion

**Figure 1:**
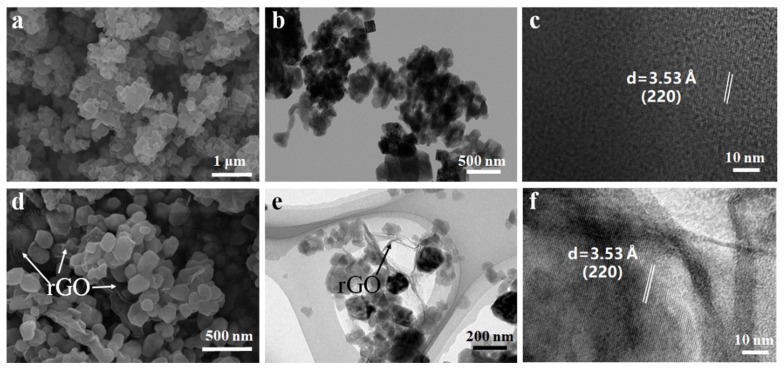
Microstructure of KCoHCP−H2−EK and KCoHCP−H2−EK/rGO: (**a**–**c**) SEM, TEM, HRTEM images of KCoHCP−H2−EK respectively; (**d**–**f**) SEM, TEM, HRTEM images of KCoHCP−H2−EK/rGO, respectively.

The microstructures of various PBA materials, especially their evolution process, were characterized by SEM and TEM, as shown in Figure 1 and Figure S1–S3. Let us first look at Figure S1a–e, both KCoHCF−CP and KCoHCF−Hx present as cubed nanoparticles, but with different sizes. Compare Figure S1a,c, the particles size of KCoHCF−CP is around 300–400 nm, which is significantly larger than KCoHCF−H2 (200 nm), and the distribution range of KCoHCF−CP particles size is more dispersed. After the potassium−eliminated treatment, the nanoparticle maintained their cubic structure, with no significant change in size. In the process of hydrothermal route to prepare KCoHCF−Hx, in order to understand the influence of hydrothermal time on the particle structure, we respectively characterized the microstructure of the comparison samples with different hydrothermal time. Figure S1a–e shows the morphology and structure of KCoHCP−Hx. The size of the samples changed with the hydrothermal reaction time. The morphology of KCoHCP−H1, obtained after the hydrothermal reaction time for 1 h is not very regular, mainly polyhedron, and the size range was 250–300 nm. When the hydrothermal reaction time became 2–4 h, the crystals began to uniform. At this time, the cubes are relatively perfect with the appearance of a very smooth surface, and the particle size becomes gradually smaller. The size of sample KCoHCP−H2 is about 200 nm. Over time, the size of sample KCoHCP−H4 is only 100nm. After the time continued to extend to 12 h, the crystal particle size was still 100 nm, this indicates that the particles stopped changing after 4 h of hydrothermal reaction. With the increase of hydrothermal time, the crystal began to grow continuously, and the larger crystal particles in the solution also began to react to form nanocrystals with a relatively regular cubic structure. Therefore, the particle size first decreased, then increased, and gradually stabilized.

The SEM images of KCoHCP−Hx (x = 1, 2, 4, and 12)−EK after depotassification were shown in Figure S1f–j. After potassium removal, the morphology of the KCoHCP−H1−EK sample can basically maintain the polyhedral structure, and the size was slightly smaller than KCoHCP−H1, about 180 nm. The morphology of KCoHCP−H2−EK could also basically maintain the cube structure. The crystal size range is 100–150 nm, which is much smaller than the size of KCoHCP−CP−EK (400 nm), which was one of the reasons for the different properties. The morphology of KCoHCP−H4−EK and KCoHCP−H12−EK samples changed to some extent, and agglomeration occurred. There was little difference in size between them, with the size range of 200–400 nm. This result shows that the hydrothermal reaction time of the precursor would affect the morphology and particle size of the samples. The basic morphology of KCoHCP−H1−EK and KCoHCP−H2−EK had not changed greatly, and the particle size has slightly decreased, which is related to the reduction of the framework of the whole KCoHCP by the removed K ions. The morphology of KCoHCP−H4−EK and KCoHCP−H12−EK samples changed greatly, the agglomeration phenomenon was obvious, there was a trend of transformation from cube to polyhedron, and the edge of grain was not clear. The reason might be that the samples themselves had a small size and did not have additional dispersant, resulting in spontaneous agglomeration during the process of depotassium.

The SEM images of the rGO composite electrodes are displayed in Figure 1 and Figure S2. Compared with KCoHCP−CP−EK and KCoHCP−H2−EK, 2D layered rGO can be observed in the KCoHCP−EK/rGO composite samples, and the layered structure features were obvious. The KCoHCP−EK/rGO composites formed a good 3D interconnected conductive network with the introduction of rGO.

The TEM results also show consistent properties, as shown in Figure 1 and Figure S3. In addition, The HR−TEM images show the presence of lattice fringes of around 3.48 Å of KCoHCP−CP−EK and 3.53 Å of KCoHCP−H2−EK corresponding the (220) crystal plane of cubic KCoHCP−EK. This result is consistent with that measured by XRD.

**Figure 2:**
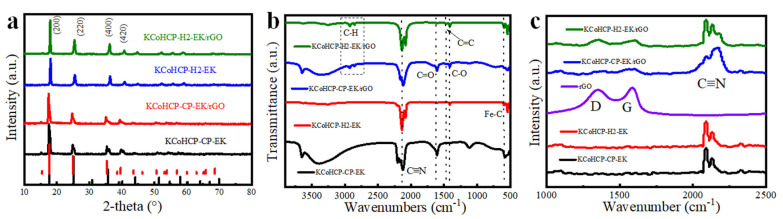
(**a**) XRD images of KCoHCP−H2−EK/rGO, KCoHCP−H2−EK, KCoHCP−CP−EK/rGO, KCoHCP−CP−EK; (**b**) FT−IR spectra images of KCoHCP−H2−EK/rGO, KCoHCP−H2−EK, KCoHCP−CP−EK/rGO, KCoHCP−CP−EK; (**c**) Raman spectra images of KCoHCP−H2−EK/rGO, KCoHCP−H2−EK, KCoHCP−CP−EK/rGO, KCoHCP−CP−EK.

The XRD diffraction patterns of KCoHCP−CP, KCoHCP−CP−EK, KCoHCP−H2, KCoHCP−H2−EK, KCoHCP−CP−EK/5%rGO, and KCoHCP−H2−EK/5%rGO were shown in Figure 2a and Figure S4. The crystal planes of KCoHCP−CP and KCoHCP−H2 in (200), (220), (400), and (420) are consistent with those of previously reported KCoFe(CN)_6_ (PDF#75−0038) and these peaks are consistent with the reported PBA−like characteristic peaks [33,34]. Peaks of KCoHCP−CP−EK and KCoHCP−H2−EK appear on the crystal planes (110) and their crystal planes (200) and (220) increase and decrease slightly, respectively, which is similar to the overall peak pattern of Co_3_[Fe(CN)_6_]_2_ (PDF#46−0947) [37]. The results of XRD confirmed the success of the potassium removal scheme. Since the existing form of rGO was amorphous and the content of rGO may be relatively low, no new diffraction peaks were found of PBA/rGO.

To further prove the existence of rGO, KCoHCP−CP−EK, KCoHCP−H2−EK, KCoHCP−CP−EK/rGO, and KCoHCP−H2−EK/rGO samples were measured by Raman and FT−IR, and the results were illustrated in Figure 2b,c, respectively. The KCoHCP−EK samples presented an intense band at 2060 cm^−1^ assigned to C≡N stretching. Bands at 3370 and 1610 cm^−1^, attributed to O−H stretching and H−O−H angular deformation modes, respectively, are also noted. These bands are associated with the presence of interstitial water in the structure of PBA. The spectrum of KCoHCO−EK/rGO indicated the presence of residual functional groups in the carbon material, showing bands corresponding to C=O (1710 cm^−1^), C=C (1565 cm^−1^), C−O (1415 cm^−1^), C−H stretching modes (2910 cm^−1^), and carboxylic acid (3780 cm^−1^). The Raman spectral data of the above four samples shows the intense bands attributed to the C≡N vibration mode of KCoHCP−CP−EK and KCoHCP−H2−EK at 2092 cm^−1^. Raman spectroscopy data for KCoHCP−CP−EK/rGO and KCoHCP−H2−EK/rGO present two characteristic bands of carbonaceous materials: the D band, centered at 1350 cm^−1^ and associated with sp^3^ C−C atoms, structural defects, and heteroatoms; and the G band (1576 cm^−1^), assigned to in−plane sp^2^ C=C stretching. In addition, the intensity ratio of the D and G bands (I_D_/I_G_) decreased from 0.93 (rGO) to 0.98 (KCoHCP−CP−EK/rGO) and 1.07 (KCoHCP−H2−EK/rGO), respectively, indicating that the KCoHCP−CP−EK/rGO and KCoHCP−H2−EK/rGO composites would introduce more defects during the synthesis, which could make the electron transport faster, thereby improving the cycling performance of the samples.

**Figure 3:**
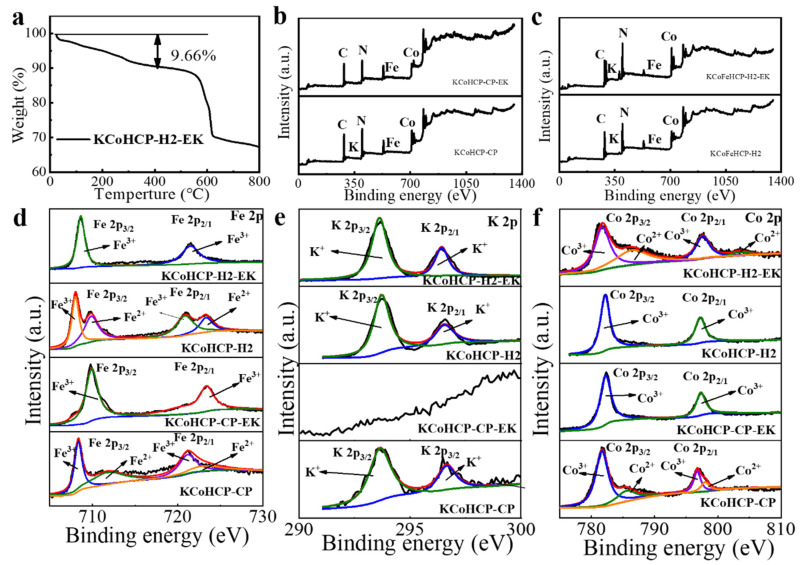
The analysis of thermogravimetric and surface chemical bonds: (**a**) The thermogravimetric curve of KCoHCP−H2−EK; (**b**–**f**) XPS spectras of KCoHCP−CP, KCoHCP−CP−EK samples and KCoHCP−H2, KCoHCP−H2−EK samples.

According to the ICP−OES analysis in Table 1, the contents of all the metal atoms are normalized to that of Fe. Thus, the chemical compositions of the as−synthesized KCoHCP−CP, KCoHCP−CP−EK, KCoHCP−H2, and KCoHCP−H2−EK materials are confirmed as K_0.9_CoFe(CN)_6_, K_0.03_Co_1.45_Fe(CN)_6_, K_1.2_CoFe(CN)_6_, and K_0.5_Co_1.2_Fe(CN)_6_. It was found that the K−ion content of the potassium−eliminated sample has decreased significantly, allowing more of the Na^+^ to enter into the lattice gap, which is beneficial for the enhancement of capacity and rate performance. In addition, as present in thermogravimetric analysis (Figure S5 and Figure 3a, respectively), the water content of KCoHCP−CP−EK and KCoHCP−H2−EK samples were 27.41% and 9.66%, suggesting the structure of K_0.03_Co_1.45_Fe(CN)_6_·6.5H_2_O and K_0.5_Co_1.2_Fe(CN)_6_·2H_2_O we obtained.

X-ray photoelectron spectroscopy (XPS) was used to determine the chemical valence and composition of the electrode in a pristine state and depotassium. Figure 3b shows the survey spectrum of pristine KCoHCP−CP and KCoHCP−CP−EK, while Figure 3d–f shows the fitting curves spectra of K 2p, Fe 2p, and Co 2p, which indicates that K, Co, and Fe elements were probed on the surface of KCoHCP−CP, and only Fe and Co elements were probed on the surface of KCoHCP−CP−EK. In addition, Figure 3d–f showed that Co(III) and Fe(II) appear in KCoHCP−CP cubes, which is ascribed to the intramolecular electron transfer from Co(II) to Fe(III), namely, partial Co(II)−N≡C−Fe(III) configurations turns to the Co(III)−N≡C−Fe(II) one [38]. Additionally, the Fe and Co ions of the KCoHCP−CP−EK were completely converted to Fe(III) and Co(III). Two peaks appear at 709.8 eV and 721.5 eV, corresponding to Fe^3+^. The two peaks at 782.2 eV and 797.4 eV are for Co^3+^. Therefore, XPS characterization can confirm the successful desorption of K ions, leaving unoccupied interstitial sites for potential sodium storage, and this conclusion was consistent with that of ICP−OES. Figure 3c shows the survey spectrum of pristine KCoHCP−H2 and KCoHCP−H2−EK, indicating that K, Co, and Fe elements were probed on the surface of KCoHCP−CP and KCoHCP−H2−EK. This result confirmed that the KCoHCP−H2−EK sample did not achieve complete potassium removal. Two main peaks could be observed at about 297.1 eV and 294.2 eV in Figure 3e, corresponding to K 2p. In addition, through the comparison of surface elements, it was found that the K content was also reduced from 9.4% to 3.6%, which also confirmed the above ICP−OES and XRD results to a certain extent. The results in Figure 3d show that in the Fe 2p3/2 spectrum of the KCoHCP−H2, the two peaks at 708.6 eV and 721.6 eV correspond to Fe^3+^. Unlike the KCoHCP−CP, there was no corresponding peak of Fe^2+^. The spectrum of Co 2p3/2 shows two valence states of Co^2+^ and Co^3+^, the peaks of Co^2+^ appear at 781.7 eV and 797.2 eV, and the peaks of Co^3+^ appear at 786.6 eV and 803.4 eV. This result also confirms the previous ICP−OES results. The ratio of K, Co, and Fe content in the hydrothermally synthesized PBA was not 1:1:1, and the valence of Co element partially changes to +3 valence state during the synthesis process. Co completely presents +3 valence, and Fe presents two valence states of +2 and +3 in KCoHCP−H2−EK. Two peaks appear at 782.1 eV and 797.3 eV, corresponding to Co^3+^. The two peaks at 707.9 eV and 720.9 eV are for Fe^3+^, and the two peaks at 709.9 eV and 723.4 eV are for Fe^2+^. This result could be attributed to incomplete depotassium, some Co ions might occupy the positions of Fe ions, so that Fe ions appear in two valence states of +2 and +3 (Figure 3d–f).

**Figure 4:**
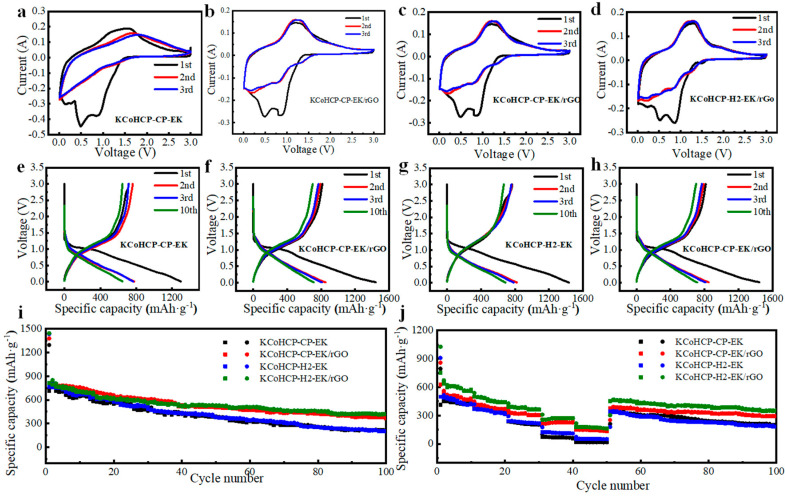
Electrochemical characterization of KCoHCP−CP−EK and KCoHCP−CP−EK/rGO: (**a**–**d**) CV curves of KCoHCP−CP−EK, KCoHCP−CP−EK/rGO, KCoHCP−H2−EK, KCoHCP−H2−EK/rGO; (**e**–**h**) the charge/discharge curves of different samples after 1st, 2nd, 3rd, and 10th cycles; (**i**) charge/discharge cycle profiles of different sample at 0.1 A g^−1^; (**j**) capacity of different samples at various current densities of 0.1, 0.2, 0.5, 1 and 2 A·g^−1^.

In view of the excellent morphology and structural characteristics of the material, we used it as anodes for SIBs, and made electrochemical sodium storage test. Cyclic voltammetry (CV) tests were performed in the voltage range of 0.01–3.0 V at a scan rate of 0.1 mV·s^−1^ by electrochemical workstation, and the results of the first three cycles were shown in Figure 4a–d. KCoHCP−CP−EK and KCoHCP−H2−EK exhibited similar features, that the peaks around 1.40/1.54 V correspond to the redox reaction of Fe^3+^/Fe^2+^ and 1.05/1.09 V correspond to the redox reaction of Fe^3+^/Fe^2+^. The reduction peak at 0.21 V could be noted during the first cycle and weakened significantly in subsequent tracing, which might be attributed to the peak of rGO. From the second cycle, two pairs of definite redox peaks appeared at 1.80/1.16 V and 1.35/1.36 V, corresponding to the peaks of Fe and Co, respectively.

Figure 4e–h showed discharge/charge profiles of the KCoHCP−EK and KCoHCP−EK/rGO anodes for the 1st, 2nd, 3rd, and 10th cycles at a constant current density of 0.1 A·g^−1^ with operating voltages window of 0.01–3.0 V. Obviously, the initial discharge of the sample demonstrates a well−defined voltage plateau at around 1.0.1d KCoHCP−EK/rGO long sloping curve down to 0.01 V and tends to be stable in subsequent cycles. The initial discharge and charge capacities of KCoHCP−CP−EK were 1296.5 mAh·g^−1^ and 717.2 mAh·g^−1^, which might result from irreversible capacity loss, containing inevitable formation of a solid electrolyte interface (SEI) [39,40,41], the decomposition of the electrolyte and the crystal water. Note that this phenomenon is common for most transition metal based anode materials. The following other samples also had the same rule. The initial discharge/charge capacities of KCoHCP−CP−EK/rGO, KCoHCP−H2−EK, and KCoHCP−H2−EK/rGO anode were 1379.2/788.2 mAh·g^−1^, 1435.5/769.6 mAh·g^−1^ and 1445.0/846.6 mAh·g^−1^, respectively. In the first five cycles, the coulombic efficiency was increased from the initial 55.30% to 97.75% (the 2nd) and 99.95% (the 10th). Moreover, the discharge/charge voltage profile of the other samples (Figure 4f−h) at a constant current density of 100 mA·g^−1^ showed almost the same characteristics as that of KCoHCP−CP−EK. The KCoHCP−CP−EK/rGO, KCoHCP−H2−EK, and KCoHCP−H2−EK/rGO samples’ coulombic efficiency increased from 57.20% to 96.50%, 52.0% to 97.30%, and 58.59% to 98.45%, respectively. Whereas, the initial discharge/charge capacity of the KCoHCP−CP−EK anode was 1296.5/717.2 mAh·g^−1^, which was smaller than that of KCoHCP−H2−EK/rGO (1445.0/846.6 mAh·g^−1^). The KCoHCP−CP−EK has more crystal water than KCoHCP−H2−EK, so the crystal water of KCoHCP−CP−EK decomposition consumed more Na^+^ contributing to a large initial discharge capacity.

The KCoHCP−Hx−EK (x = 1, 2, 4, and 12) with different hydrothermal reactions (represented by x) time were also used as anodes of SIBs to characterize their electrochemical performance. As shown in Figure S6, the cycling performance of KCoHCP−Hx−EK was tested at a current density of 0.1 A·g^−1^ and the initial discharging specific capacities of the samples were above 1000 mAh·g^−1^. The Coulombic efficiency of KCoHCP−H1−EK, KCoHCP−H2−EK, KCoHCP−H4−EK, and KCoHCP−H12−EKs was 53.6%, 52.0%, 45.2% and 35.4%, respectively, corresponding to the irreversible generation of SEI films after the first discharge process. Obviously, the cycling performance of KCoHCP−H2−EK was better than that of other samples. Capacity retentions of KCoHCP−H1−EK, KCoHCP−H2−EK, KCoHCP−H4−EK, and KCoHCP−H12−EK, were 17.2%, 35.6%, 29.6%, and 21.2%, respectively. It can also be seen from the SEM and TEM images that the KCoHCP−H2−EK sample has the smallest grain size and less agglomeration than the samples prepared with other hydrothermal reaction times. Compared with KCoHCP−H2−EK, which capacity retentions were 35.6% after 100 cycles, the cycling performance of KCoHCP−CP−EK, KCoHCP−CP−EK/rGO, and KCoHCP−H2−EK/rGO were also studied and their capacity retentions were 27.8%, 46.5%, and 58.2%, after 100 cycles (Figure 4i). The performance of KCoHCP−H2−EK was superior to that of KCoHCP−CP−EK, which was attributed to the lower crystal water content and smaller grain size of the KCoHCP−H2−EK sample. Moreover, compared with the prisine KCoHCP−EK samples, the capacity retention of the samples doped with rGO were improved to a certain extent, which showed that the introduction of rGO could effectively improve the cyclic properties of KCoHCP−EK/rGO composites. At the same time, it can be clearly seen from the trend of the curve that the capacity fading phenomenon of the composite samples were alleviated compared with pristine samples, which had a significant inhibitory effect on the capacity fading.

Figure S7 shows rate performance of KCoHCP−Hx−EK (x = 1, 2, 4, and 12) at various current densities. Compared with KCoHCP−CP−EK and other samples with hydrothermal reaction time, the performance of KCoHCP−H2−EK was better. The stable discharge specific capacity of KCoHCP−H2−EK were 428.1, 325.4, 219.2, 111.1, and 51.0 mAh·g^−1^ at current densities of 0.1, 0.2, 0.5, 1, and 2 A·g^−1^, respectively. The capacities decrease regularly with increase of cyclic rates, but once the current density returns to 0.1 A·g^−1^, the capacity also recovers to the initial level, indicating an excellent rate capability even after continuously cycled at high rates. The excellent rate capability of KCoHCP−H2−EK anode was attributed to its lower contact resistance and charge transfer resistance and faster sodium ion diffusion rate as its active material. It is evident from Figure 4j that KCoHCP−EK/rGO composites also exhibit excellent rate capability. The stable discharge specific capacity of KCoHCP−H2−EK/rGO were 558.9, 441.7, 363.9, 271.1, and 162.1 mAh·g^−1^ at current densities of 0.1, 0.2, 0.5, 1, and 2 A·g^−1^, respectively, which was higher than that of the pristine sample. Meanwhile, the stable discharge specific capacity of KCoHCP−CP−EK/rGO were 467.2, 369.1, 304.1, 226.6, and 135.5 mAh·g^−1^ at current densities of 0.1, 0.2, 0.5, 1, and 2 A·g^−1^, respectively, which was also higher than the pristine sample. This result indicates that the composite electrode material had good reversible performance after charge−discharge cycles at different current densities. KCoHCP−H2−EK/rGO exhibited the best electrochemical performance. The observed results demonstrate an obvious improvement in electrochemical performance of KCoHCP−EK/rGO composites electrode, especially in specific capacity and rate capability. Owing to the rGO, the composites can form a well−connected charge−conducting network, which improves the cycling stability and reaction kinetics of the electrode, and can buffer the large volume change during cycling to optimize the electrochemical performance [41,42].

**Figure 5:**
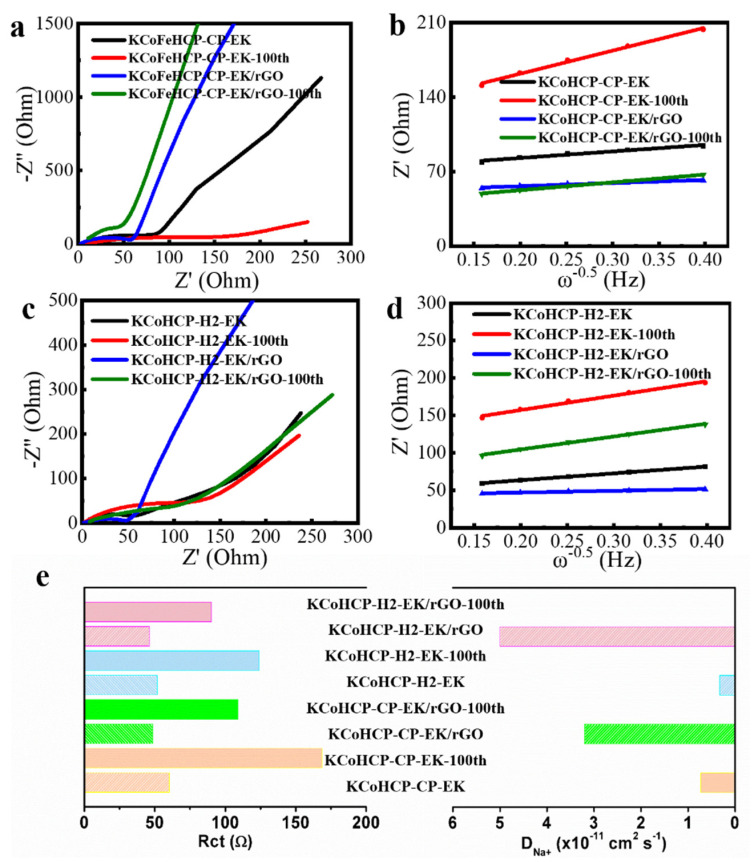
(**a**,**c**) Nyquist plots of different samples; (**b**,**d**) the liner fitting between Z′ and ω^−0.5^ at low−frequency region of different samples; (**e**) the calculated *R_ct_* and *D_Na+_* values.

In order to further investigate the interfacial properties and comparing the electrode kinetic reactions, electrochemical impedance spectroscopy (EIS) was performed for pristine KCoHCP−CP−EK, KCoHCP−H2−EK, KCoHCP−CP−EK/rGO, and KCoHCP−H2−EK/rGO. After 100 cycles, the resultant samples (named as KCoHCP−CP−EK−100, KCoHCP−H2−EK−100, KCoHCP−CP−EK/rGO−100, and KCoHCP−H2−EK/rGO−100), exhibit the EIS results of the four electrodes in Figure 5. The obtained Nyquist plots consists of a semicircle in the high frequency region and an oblique line in the low frequency region. The high frequency circle is connected with the resistance of electrolyte resistance (*R_s_*), the other semicircle at high−to−medium frequencies is assigned to the charge transfer resistance (*R_ct_*), the Warburg impedance (*W_o_*), which reflects the diffusion rate of Na^+^. The results of corresponding interfacial resistances values by fitting the spectra of equivalent circuit are summarized. According to the fitting parameters, *R_ct_* values were 60.4 Ω (KCoHCP−CP−EK), 51.8 Ω (KCoHCP−H2−EK), 48.5 Ω (KCoHCP−CP−EK/rGO), and 46.3 Ω (KCoHCP−H2−EK/rGO), respectively. The *R_ct_* value of KCoHCP−CP−EK−100, KCoHCP−H2−EK−100 KCoHCP−CP−EK/rGO−100, and KCoHCP−H2−EK/rGO−100, increased to 168.6, 124.0, 108.9, and 90.1 Ω. The result indicates that the addition of rGO could greatly suppress the side reactions between anode materials and organic electrolyte, hindering the increase of *R_ct_* resistance value. It is also shown that the composite samples had the faster charge transfer ability, and this result was also reflected in their respective electrochemical properties. The sodium ion diffusion coefficient (*D_Na+_*) in the solid active particles can be calculated according to the formula [43]:

Z′ = R_s_ + R_ct_+ σω^−1/2^ (1)

D_Na_^+^ = R^2^T^2^/2A^2^n^4^F^4^C^2^σ^2^ (2)

where R is gas constant, T is room temperature in experiment, F is the Faraday constant, A represents the electroactive area of cathode material, n is the number of transferred electrons in the reaction, C is concentration of lithium ion in the material, and σ is the Warburg coefficient, which is the slope of the line between Z′ and ω^−1/2^ in Equation (1). It is calculated from the slope fitting in the low−frequency region. The fitting graph obtained is shown in Figure 5b,d. The calculated *D_Na+_* values were shown in Figure 5e. The *D_Na+_* values of the KCoHCP−CP−EK, KCoHCP−H2−EK, KCoHCP−CP−EK/rGO, and KCoHCP−H2−EK/rGO were calculated to be approximately 7.3 × 10^−12^, 3.2 × 10^−11^, 3.3 × 10^−1^, and 5.0 × 10^−11^ cm^2^·S^−1^, respectively. Therefore, the results showed that the addition of rGO could make the anode material had faster ions and electrons transfer ability, reduce the charge transfer resistance, and improve the electrochemical ability.

From the comparison of electrochemical performance data, KCoHCP−CP−EK has an initial charging capacity of 717.2 mAh·g^−1^, an initial discharge capacity of 1296.5 mAh·g^−1^, an initial efficiency of 55.3%, and a capacity retention rate of 27.8% after 100 cycles with 0.1 A·g^−1^ current density. KCoHCP−H2−EK showed better initial charge−discharge capacity and cycle performance. The initial charge capacity of the half battery is 769.6 mAh·g^−1^, the initial discharge capacity is 1435.5 mAh·g^−1^, the initial efficiency is 53.6%, and the capacity retention rate of 0.1 A·g^−1^ after 100 cycles is 35.6%. This is mainly attributed to the smaller particle size and less crystalline water content obtained by hydrothermal method. The comparison of the specific capacity, the cycling capability of KCoHCP−EK/rGO and KCoHCP−H2−EK/rGO samples to those of other PBA anode materials in the literature are shown in Table 2. It can be clearly seen that our PBA based SIB anodes show the best initial charge−discharge specific capacity compared with some of other PBA anode materials, hard carbon [44,45,46], transition metal oxide materials [47,48,49,50,51] and alloy materials based anodes [52,53]. It should be noticed that either for KCoHCP−EK/rGO or KCoHCP−H2−EK/rGO anode, the initial efficiency is relatively low, and their cyclic performance decay is relatively fast. It is well known that the residual of water in the anode has a great influence to the sodium ion batteries performance. The decomposition of the residual water at high potential will produce gases, and the residual water has a great influence on the by−reactants and electrolytes as well. In our work, according to the TGA data, the moisture content of the KCoHCP−EK samples prepared by the precipitation method is significantly higher than that prepared by the hydrothermal method. Therefore, the cycle life of KCoHCP−H2−EK samples is higher than that prepared by the precipitation method. According to thermogravimetric analysis, it needs to heat up to 400 °C to completely remove the residual crystal water in the as synthesized PBA based anodes. Since we have not conducted the high temperature heat treatment on the materials, which could be an important reason for the deterioration of the cycle performance of our sample, which is also the place we need to improve in the subsequent work.

## 4. Conclusions

In summary, K_x_Co_1.5−0.5x_Fe(CN)_6_ and K_x_Co_1.5−0.5x_Fe(CN)_6_/rGO have been synthesized by an economical and scalable method. The influence of different precursor preparation route and the introduction of rGO has been explored. The initial charge/discharge capacities of KCoHCP−CP−EK/rGO were 788.2/1379.2 mAh·g^−1^. Compared with KCoHCP−CP−EK, the capacity retention rate of 100 cycles was increased from 27.8% to 46.5%. The best performance sample of KCoHCP−H2−EK/rGO showed an initial charge/discharge capacities as high as 846.7/1445.0 mAh·g^−1^ and the capacity retention rate of 100 cycles increased from 35.6% to 58.2%. In terms of rate discharge performance, KCoHCP−CP−EK/rGO sample exhibited the capacity of 226.6 and 135.5 mAh·g^−1^ at a relatively high density of 1 and 2 A·g^−1^, respectively. At the mean time, the KCoHCP−H2−EK/rGO sample exhibited a highly reversible capacity of 271.1 and 162.1 mAh·g^−1^ at a high density of 1 and 2 A·g^−1^, respectively. Compare to some of other SIB anode materials, it can be clearly seen that our PBA show the best initial charge−discharge specific capacity. The improved performance of the KCoHCP−H2−EK/rGO can be attributed to the unique double−active site structure of PBA and the synergistic effect of the PBA/rGO composites, which enhanced the electrical conductivity of the active materials and alleviated the collapse of the active material during cycling. It is believed that KCoHCP−EK, KCoHCP−H2−EK, KCoHCP−EK/rGO, and KCoHCP−H2−EK/rGO, will have relatively great commercial application potential once the problems of first efficiency and circulation could be overcome. Consequently, this research project provides an effective way for the development of SIBs anode materials.

## Figures and Tables

**Table 1:** Results of ICP−OES measurements of three element (Co, Fe, K) weight percentage in KCoHCP−CP, KCoHCP−CP−EK, KCoHCP−H2, and KCoHCP−H2−EK.

	Co	Fe	K	Other
KCoHCP−CP	12.7%	12.9%	11.4%	63%
KCoHCP−CP−EK	23.7%	16.3%	0.6%	59.4%
KCoHCP−H2	11.6%	12.0%	14.5%	50.3%
KCoHCP−H2−EK	18.2%	16.2%	8.2%	57.4%

**Table 2:** Cycle stability of PBA anode materials.

Samples	Electrode Type	Cycling Properties				Ref.
		Current Density (A·g^−1^)	Specific Capacity of Initial Discharge (mAh·g^−1^)	Cycle Number	Specific Capacity after Cycling (mAh·g^−1^)	
K_0.03_Co_1.45_Fe(CN)_6_/5%rGo	SIBs anode	0.1	1379.2	100	308.57	This work
KCoFe(CN)_6_(H2)−EK/5%rGO	SIBs anode	0.1	1444.5	100	420.54	This work
FeHCF	SIBs anode	0.6	80.0	250	68.8	[22]
CrCr−PBA	SIBs anode	0.5	108.2	100	101.1	[23]
K_1−x_Fe_2+x/3_(CN)_6_	LIBs anode	0.0875	450.0	40	375.5	[24]
Co_3_[Co(CN)_6_]_2_	LIBs anode	0.02	566.2	5	304.0	[25]
KMnHCF	LIBs anode	0.05	777.0	50	485.0	[37]
Hard carbon spheres	SIBs anode	0.1	301	500	181	[44]
Biomass derived hard carbon	SIBs anode	0.02	413	500	320	[45]
Orange peel derived hard carbon	SIBs anode	0.07	170	100	125	[46]
TiO_2_/MWCNTs	SIBs anode	0.1	477	140	216	[47]
Double−carbon enhanced TiO_2_ nanotubes	SIBs anode	1	164	500	135.8	[48]
TiO_2_/C	SIBs anode	1	180	1600	175	[49]
3D Sb–Bi alloy/N−doped porous carbons (N–PCs)	SIBs anode	10	318.3	6000	215.2	[52]
Bi_0.75_Sb_0.25_ alloy	SIBs anode	2.5	335	2000	291	[53]

## Supplementary Materials

The following supporting information can be downloaded at: https://www.mdpi.com/article/10.3390/nano13020264/s1. Figure S1: SEM images of (a) KCoHCP−CP; (b) KCoHCP−H1; (c) KCoHCP−H2; (d) KCoHCP−H4; (e) KCoHCP−H12; (f) KCoHCP−CP−EK; (g) KCoHCP−H1−EK; (h) KCoHCP−H2−EK; (i) KCoHCP−H4−EK; (k) KCoHCP−H12−EK; Figure S2: SEM images of (a) KCoHCP−CP−EK; (b–c) KCoHCP−CP−EK/rGO; (d) KCoHCP−H2−EK; (e–f) KCoHCP−H2−EK/rGO. Figure S3: (a) TEM image of KCoHCP−CP−EK and (b) HRTEM image of KCoHCP−CP−EK. Figure S4: XRD patterns of KCoHCP−CP, KCoHCP−CP−EK, KCoHCP−H2 and KCoHCP−H2−EK. Figure S5: The thermogravimetric curve of KCoHCP−CP−EK. Figure S6: The cycling performance of KCoHCP−Hx−EK at a current density of 0.1 A·g^−1^. Figure S7: The rate performance of KCoHCP−Hx−EK.
