# Supplementary material for: KxCo1.5−0.5xFe(CN)6/rGO with Dual−Active Sodium Ion Storage Site as Superior Anode for Sodium Ion Battery
Source: Nanomaterials (Basel). 2023 Jan 7;13(2):264. doi: 10.3390/nano13020264 (PMC9865383; doi:10.3390/nano13020264)
Supplement: Supplementary file 1 [file nanomaterials-13-00264-s001.zip › nanomaterials-2125491-supplementary.pdf]

$K_xCo_{1.5-0.5x}Fe(CN)_6$/rGO with Dual−Active Sodium Ion Storage Site as Superior Anode for Sodium Ion Battery

Gang Zhou [1,*], Mincong Fan [1,2], Lei Wang [2], Xianglin Li [3,*], Danqing Liu [2] and Feng Gao [1]

[1] School of Environment and Civil Engineering, DongGuan University of Technology, Dongguan 523808, China
[2] College of Materials Science and Engineering, Shenzhen University, Shenzhen 518061, China
[3] School of Physics and Chemistry, Hunan First Normal University, Changsha 410205, China
* Correspondence: hhzhougang@163.com (G.Z.); lixl@idtu.cn (X.L.)

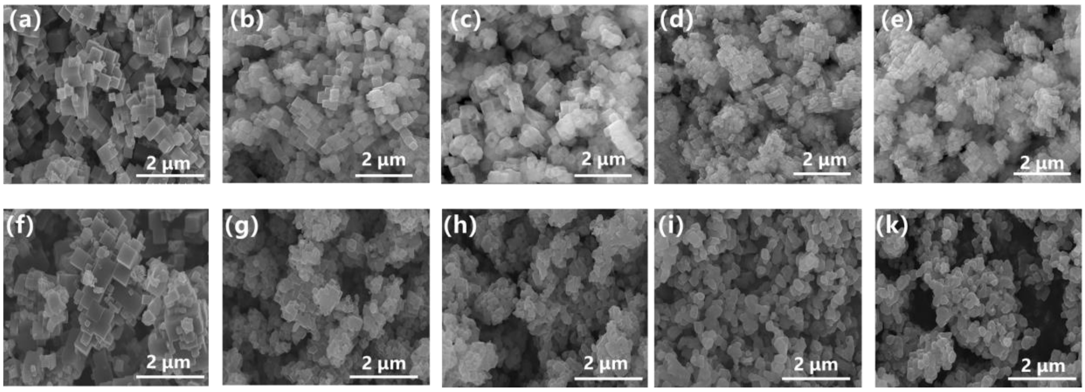

Figure S1. SEM images of (a) KCoHCP-CP; (b) KCoHCP-H1; (c) KCoHCP-H2; (d) KCoHCP-H4; (e) KCoHCP-H12; (f) KCoHCP-CP-EK; (g) KCoHCP-H1-EK; (h) KCoHCP-H2-EK; (i) KCoHCP-H4-EK; (k) KCoHCP-H12-EK;

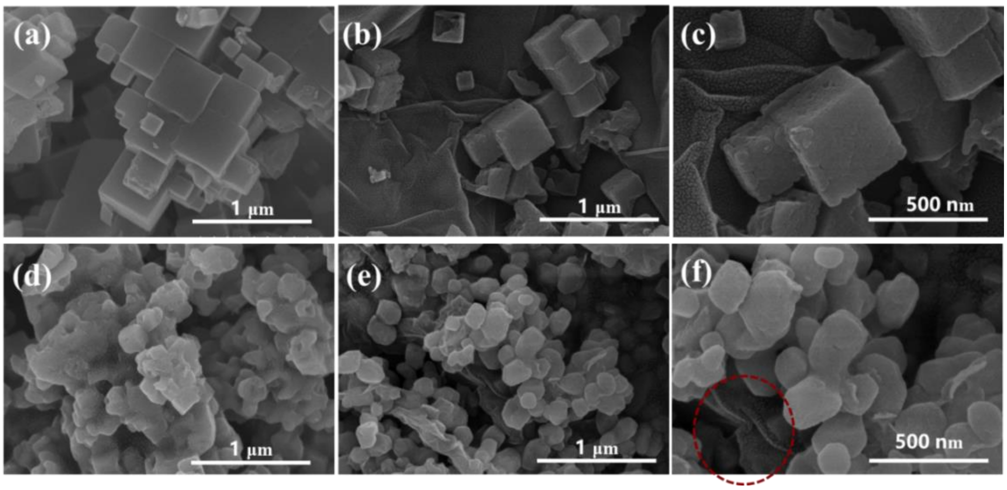

Figure S2. SEM images of (a) KCoHCP-CP-EK; (b-c) KCoHCP-CP-EK/rGO; (d) KCoHCP-H2-

EK; (e-f) KCoHCP-H2-EK/rGO

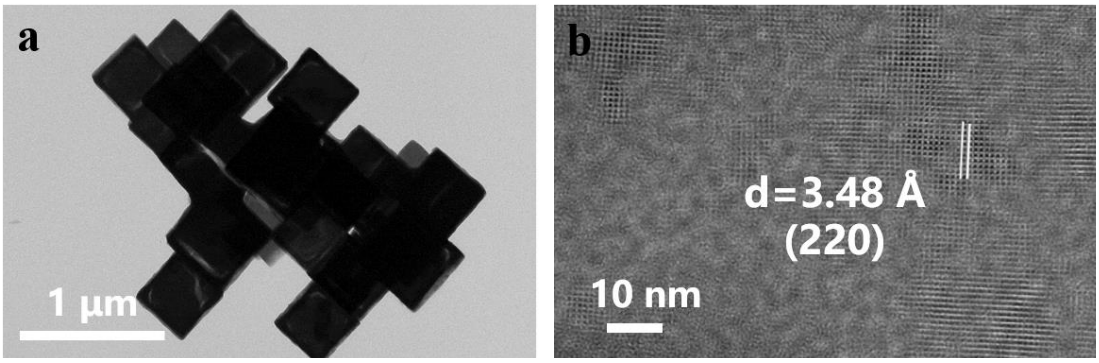

Figure S3. (a) TEM image of KCoHCP-CP-EK and (b) HRTEM image of KCoHCP-CP-EK.

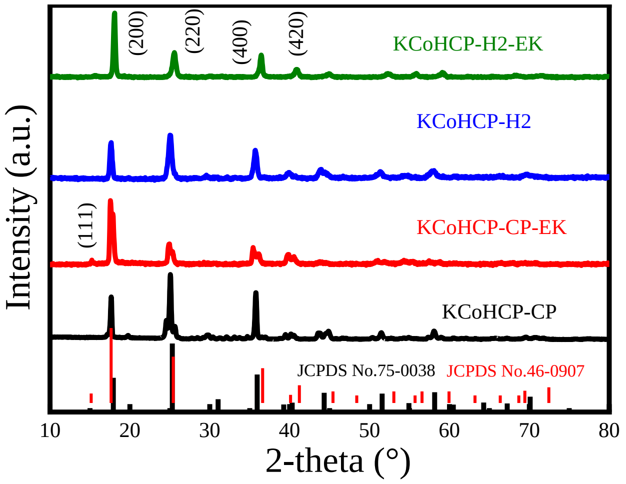

Figure S4. XRD patterns of KCoHCP-CP, KCoHCP-CP-EK, KCoHCP-H2 and KCoHCP-H2-EK

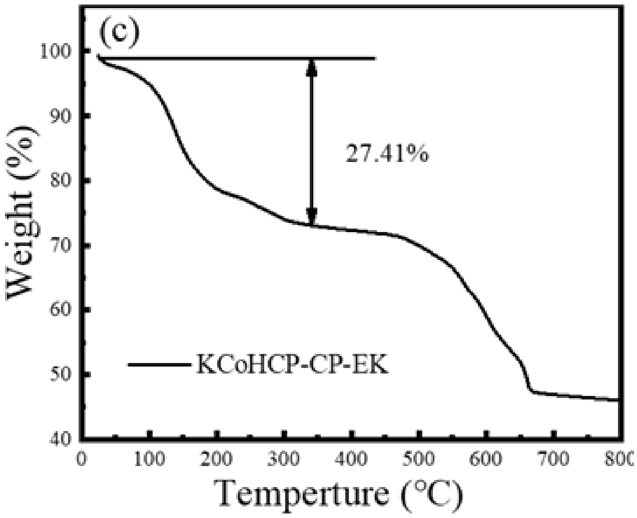

Figure S5. The thermogravimetric curve of KCoHCP-CP-EK

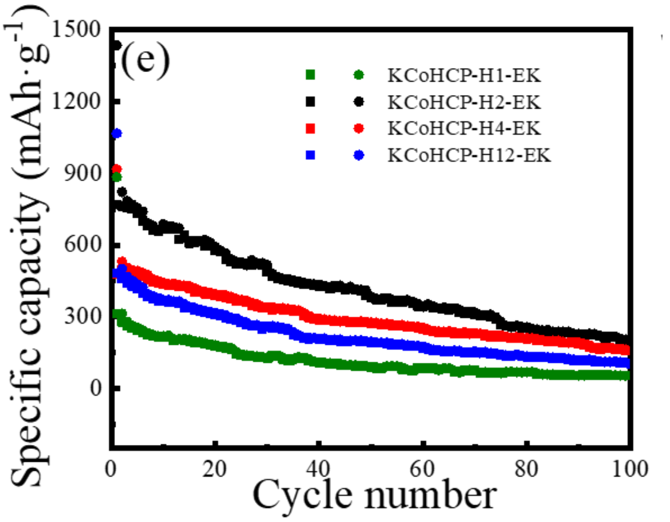

Figure S6. The cycling performance of KCoHCP-Hx-EK at a current density of 0.1 $A{\cdot}g^{-1}$.

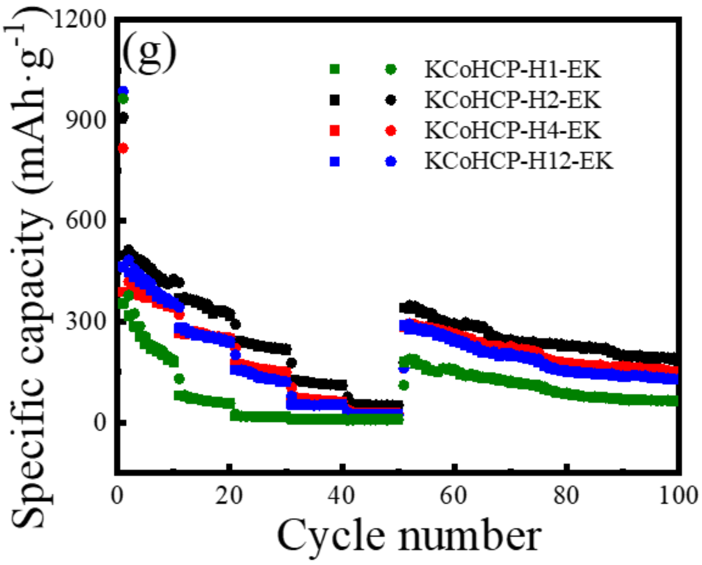

Figure S7. The rate performance of KCoHCP-Hx-EK.
